# Ultrasonic Spray Pyrolysis Synthesis and Photoluminescence of LuAG:Ce Thin Films

**DOI:** 10.3390/ma15072577

**Published:** 2022-03-31

**Authors:** Tingting Wang, Bingguo Xue, Hao Cui, Yingying Zhang, Manmen Liu, Jialin Chen, Ming Wen, Wei Wang, Xudong Sun, Shaohong Liu

**Affiliations:** 1 Key Laboratory for Anisotropy and Texture of Materials (Ministry of Education), School of Materials Science and Engineering, Northeastern University, Shenyang 110819, China; wangtingting@stumail.neu.edu.cn (T.W.); 2190017@stu.neu.edu.cn (B.X.); 13840506362@163.com (Y.Z.); wangw@atm.neu.edu.cn (W.W.); 2 State Key Laboratory of Advanced Technologies for Comprehensive Utilization of Platinum Metals, Kunming Institute of Precious Metals, Kunming 650106, China; cuihao@ipm.com.cn (H.C.); lmm@ipm.com.cn (M.L.); cjl@ipm.com.cn (J.C.); wen@ipm.com.cn (M.W.); 3 Foshan Graduate School, Northeastern University, Foshan 528311, China; xdsun@mail.neu.edu.cn; 4 State Key Laboratory of Rolling and Automation, Northeastern University, Shenyang 110819, China

**Keywords:** LED, ultrasonic spray pyrolysis, film, LuAG

## Abstract

LuAG:Ce (Lu_3_Al_5_O_12_:Ce) is one of the most important color converters in white lighting industry. Especially, LuAG:Ce film attracts more attention due to the outstanding advantages, such as the efficient heat dissipation, the saving of rare earth, and so on. Here, LuAG:Ce film on sapphire was successfully prepared by the ultrasonic spray pyrolysis process. The phase, microstructure and photoluminescence of LuAG:Ce films were investigated. LuAG:Ce films had a thickness of around 5 μm, which were well crystallized at 1000 °C in air atmosphere to form the typical garnet structure. Under the protection of CO atmosphere, increasing the annealing temperature greatly enhanced the photoluminescence performance. After annealing at 1500 °C for 5 h in CO atmosphere, 3.0 mol.% Ce^3+^ doped LuAG:Ce film exhibited the highest emission and excitation intensity. The emission intensity of 3.0 mol.% Ce^3+^ doped LuAG:Ce film annealed at 1500 °C in CO atmosphere increased up to five times, when compared with the best LuAG:Ce film annealed at 1000 °C in air atmosphere. The effects of Ce^3+^ doping concentration on the photoluminescence were also examined. As the Ce^3+^ doping concentration increased from 0.2 mol.% to 7.0 mol.%, the color of LuAG:Ce films changed from yellowish green to greenish yellow. When coupling the 3.0 mol.% Ce^3+^ doped LuAG:Ce film with a 0.5 W 450 nm blue laser, the formed device successfully emitted white light.

## 1. Introduction

Phosphor-converted white light-emitting diodes (LEDs), as the new generation solid lighting device, have been widely used in the field of display backlighting, streetlamps, headlamps and indoor illuminations, due to their high efficiency, long lifetime and energy conservation features [1,2,3,4,5]. LEDs are the most popular solid lighting device, which are composed of blue GaN chips and yellow YAG:Ce phosphor dispersed in organic resin [6,7,8]. However, organic resin or silicone has clear disadvantages, such as the extremely low thermal conductivity (~0.1 W m^−1^ K^−1^), and the thermal-induced drop in lighting efficiency [3,9,10]. Therefore, to develop high power and high efficiency LEDs, phosphor-converted materials, with high conversion efficiency, high thermal conductivity and excellent thermal stability, are the most urgent pursuit in the field of phosphor research and display industry [11,12,13].

Phosphor ceramics are thought to be the favorable choices to replace phosphor-in-silicon color converters [14,15]. LuAG:Ce ceramics not only have a broad emission band between 500 nm and 700 nm, but also possess excellent thermal stability and high luminous efficiency, which are regarded as the most promising color converters for high-power LEDs [16,17,18,19,20]. Xu et al. synthesized LuAG:Ce translucent ceramics (TC) by spark plasma sintering technique [20]. The obtained LuAG:Ce ceramics have wonderful thermal conductivity (6.3 W m^−1^ K^−1^) and high reliability (1.9% decrease after 1000 h at 85 °C and 85% humidity) [20]. Zhang et al. prepared Al_2_O_3_-LuAG:Ce composite ceramic phosphor (CCP) by vacuum solid-state sintering method [17]. They found that Al_2_O_3_ greatly improves the CCP’s thermal conductivity (18.9 W m^−1^ K^−1^) and thermal stability (the luminescence loss of only 3.2% at 200 °C) [17]. Clearly, LuAG:Ce ceramics are the excellent color converters. However, for use as the color converters, LuAG:Ce ceramics must be carefully machined and polished to the thickness of around 0.2 mm. During the time-consuming cutting and polishing processes, a large amount of LuAG:Ce is wasted.

To solve the problem, LuAG:Ce film on sapphire is a wonderful choice, which effectively combines the high thermal conductivity of sapphire and the high luminous efficiency of LuAG:Ce phosphor. The synthesis methods of film mainly include the sol-gel method [21], ultrasonic spray pyrolysis method [22], and liquid phase epitaxy (LPE) growth technology [23]. This work aims to prepare LuAG:Ce film on sapphire by the ultrasonic spray pyrolysis method. Sapphire is chosen as the substrate due to its high thermal conductivity (~30 W m^−1^ K^−1^) [24]. LuAG:Ce film on sapphire substrate can be directly used as the color converter, without needing the extra cutting and polishing process. LuAG:Ce films with different Ce^3+^ doping concentrations were prepared. Their phase compositions, microstructure and photoluminescence properties were investigated.

## 2. Materials and Methods

### 2.1. Synthesis of LuAG:Ce Films

**Figure 1:**
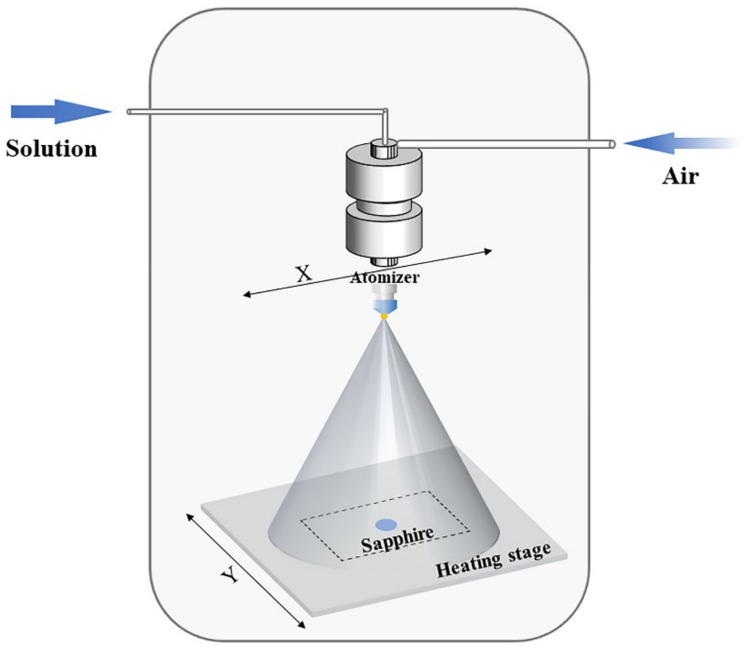
Experimental setup used for ultrasonic spray deposition.

The chemicals used were Lu_2_O_3_, Al(NO_3_)_3_·9H_2_O, Ce(NO_3_)_3_·6H_2_O and HNO_3_. All chemical reagents were of analytical grade, which were purchased from Sinopharm Chemical Reagent Corporation (Shenyang, China) and used as received. The solution of Lu(NO_3_)_3_ was prepared by dissolving Lu_2_O_3_ with proper amount of nitric acid. The spray solution was composed of Lu(NO_3_)_3_, Ce(NO_3_)_3_ and Al(NO_3_)_3_. The molar ratio of rare earth to Al was 3:5, and the total concentration of rare earth ions was 0.01 mol/L. 3 wt.% PVP was added as the surfactant. To investigate the effect of Ce^3+^ doping concentration on the photoluminescence, the spray solutions with different Ce^3+^ concentrations (0.2–7.0 mol.%) were prepared. The sapphire substrates (diameter of 20 mm, Beijing Xintian Borui Photoelectric Technology Co., Ltd., Beijing, China) were ultrasonically cleaned with deionized water, acetone and ethanol. The experimental setup for ultrasonic spray deposition was shown in Figure 1. The spray atomizer and an ultrasonic generator (UAC120, 120 K) were purchased from Cheersonic Ultrasonics Equipments Co., Ltd., Hangzhou, China. The atomizer controlled the average aerosol droplet size, which was fixed on the X-axis of a motion platform. The spray solution and a director gas were introduced into the atomizer. This director gas controlled the velocity of the droplets to arrive at the heated substrate. The distance between the atomizer and substrate was fixed to 140 mm. The flow rate of director gas, air in this case, was maintained at 2 L/min. The liquid feed rate was 1.5 mL per minute. The heating stage was controlled at 145 °C and fixed on the Y-axis of a motion platform. During the spray process, the atomizer and heating stage moved along the X-axis and Y-axis of the motion platform, respectively. The deposition time for each LuAG:Ce film was 15 min.

### 2.2. Characterization Techniques

The phase compositions of thin films were determined by X-ray diffraction (XRD, Panalytical, Almelo, The Netherlands), utilizing Cu-Kα radiation (1.54056 Å). The microstructure was observed by scanning electron microscopy (SEM, JSM-7001F, JEOL, Tokyo, Japan). The photoluminescence (PL) and photoluminescence excitation (PLE) were analyzed at room temperature using an FP-8600 fluorospectrophotometer (Jasco, Tokyo, Japan) equipped with a 60 mm diameter integrating sphere (Model ISF-834, Jasco) and a 150 W Xe-lamp for excitation. The thickness of films was measured by a stylus profiler (DEKTAK 150, Veeco, New York, NY, USA).

## 3. Results and Discussion

### 3.1. Phase Evolution of LuAG:Ce Films

**Figure 2:**
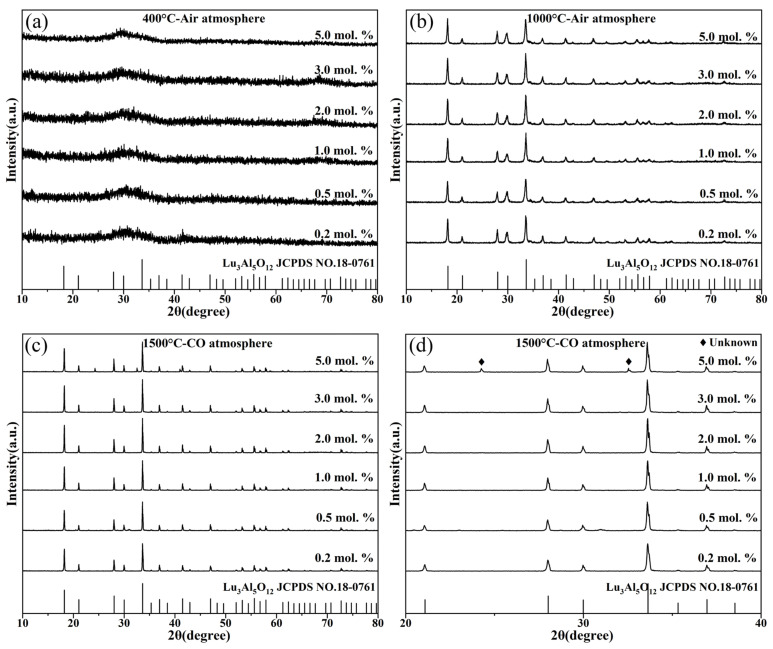
The effects of annealing temperature, annealing atmosphere and Ce^3+^ contents on the phase compositions of LuAG:Ce films: (**a**) 400 °C air atmosphere, (**b**) 1000 °C air atmosphere, (**c**) 1500 °C CO atmosphere, (**d**) enlarged XRD patterns around 2θ of 30°.

The effects of annealing temperature, annealing atmosphere and Ce^3+^ contents on the phase compositions of LuAG:Ce films were investigated. The results are shown in Figure 2. After annealing at 400 °C for 5 h in air atmosphere, the films are all amorphous substances, as shown in Figure 2a. Increasing the annealing temperature to 1000 °C, all films crystallized and exhibited the diffraction patterns of cubic Lu_3_Al_5_O_12_ (PDF#18-0761). Upon annealing below 1000 °C in air atmosphere, the Ce^3+^ doping concentration has no significant influence on the phase compositions of films. Besides, it is known that Ce^3+^ can be easily oxidized to Ce^4+^, resulting in the decrease in luminescence intensity. In order to improve the luminous intensity, LuAG:Ce films were further annealed at 1500 °C for 5 h in CO atmosphere. Their XRD patterns are shown in Figure 2c. It can be seen that after annealing at 1500 °C for 5 h in CO atmosphere, the films are still composed of cubic Lu_3_Al_5_O_12_ (PDF#18-0761) phase, and the crystallinity of films is significantly improved, as evidenced by the sharp diffraction peaks. In addition, Ce^3+^ (1.143 Å) has a larger radius than Lu^3+^ (0.977 Å). Therefore, introducing Ce^3+^ into the lattice of Lu_3_Al_5_O_12_ increases the interplanar spacing. According to the Bragg equation, the strongest diffraction peak would shift to the lower 2*θ* side. Figure 2d shows the enlarged diffraction patterns including the strongest diffraction peaks. As can be seen, the strongest diffraction peak shifts from 33.62° to 33.59° as the Ce^3+^ doping concentration increases from 0.2 mol.% to 5.0 mol.%. Due to the large amount of Ce^3+^ was incorporated into the Lu_3_Al_5_O_12_ lattice, unknown phase was observed, as shown in Figure 2d.

### 3.2. The Microstructure of LuAG:Ce Films

**Figure 3:**
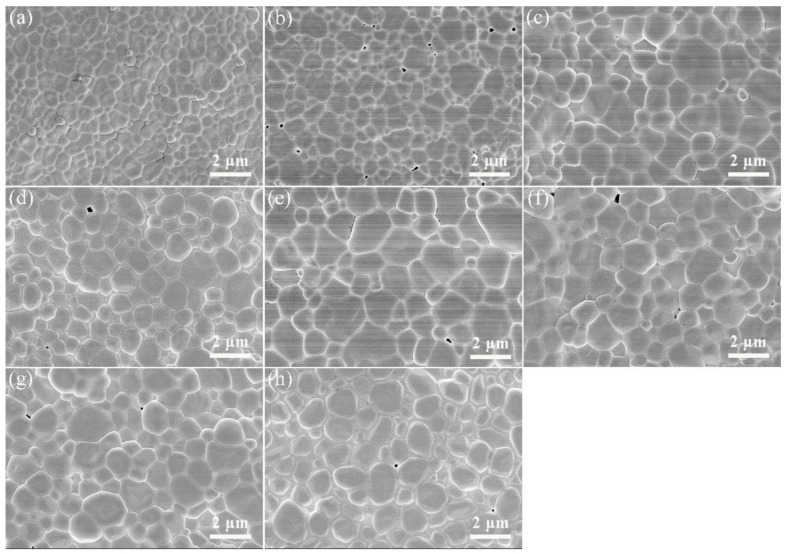
FE-SEM images of the 1500 °C CO atmosphere-annealed LuAG:Ce films with different Ce^3+^ doping concentrations: (**a**) 0.2 mol.%, (**b**) 0.5 mol.%, (**c**) 1.0 mol.%, (**d**) 1.5 mol.%, (**e**) 2.0 mol.%, (**f**) 3.0 mol.%, (**g**) 5.0 mol.%, (**h**) 7.0 mol.%.

The FE-SEM images of 1500 °C CO atmosphere-annealed LuAG:Ce films with different Ce^3+^ doping concentrations are shown in Figure 3. All the films show a flat and dense microstructure. Grain boundaries are clearly observed. A few pores are found, which situate at the triangular grain boundary region. Ce^3+^ doping concentration has significant influence on the microstructure and grain shape. For the Ce^3+^ doping concentration of 0.2 mol.%, 0.5 mol.% and 1.0 mol.%, the average grain size is 400 nm, 650 nm and 850 nm, respectively. Obviously, Ce^3+^ doping significantly improves the grain growth. The average grain size is even up to 1.0 μm when 2.0 mol.% Ce^3+^ was doped. The grain shape is also affected by the Ce^3+^ doping. As the Ce^3+^ doping concentration increases to above 1.5 mol.%, the grains become rounded. The 7.0 mol.% Ce^3+^ doped film exhibits a peculiar microstructure, whose crystal grains are rounded and isolated.

### 3.3. The Photoluminescence Properties of LuAG:Ce Films

**Figure 4:**
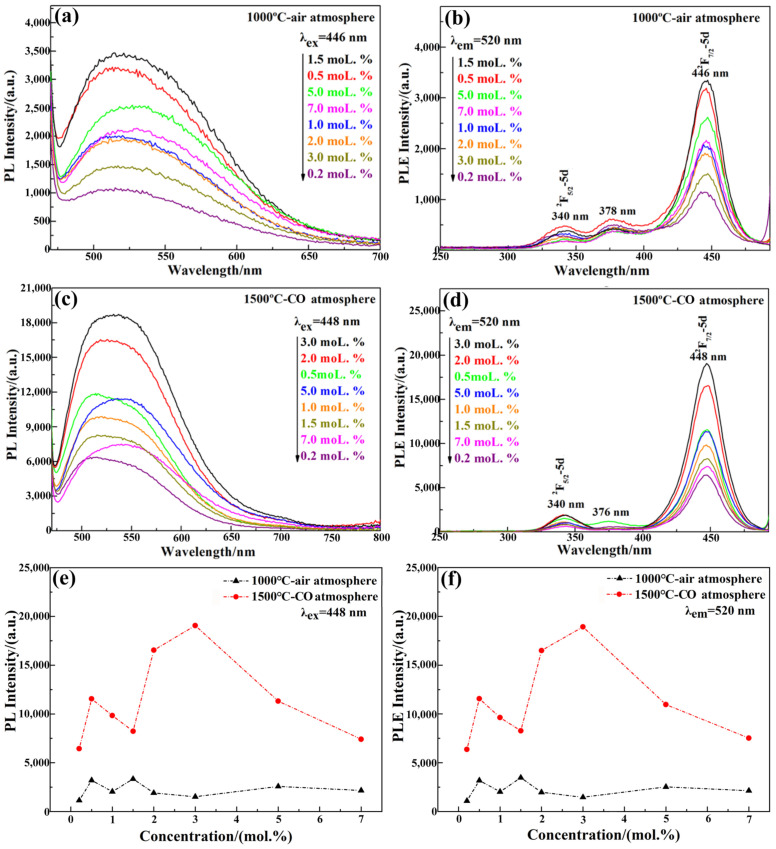
Photoluminescence properties of LuAG:Ce films with Ce^3+^ doping concentration ranging from 0.2 mol.% to 7.0 mol.%: (**a**) 1000 °C PL spectra, (**b**) 1000 °C PLE spectra, (**c**) 1500 °C PL spectra, (**d**) 1500 °C PLE spectra, (**e**) PL intensity vs. Ce^3+^ concentration, (**f**) PLE intensity vs. Ce^3+^ concentration.

The annealing temperature and atmosphere significantly influence the photoluminescence properties of LuAG:Ce films. The photoluminescent spectra of LuAG:Ce films were measured at room temperature, as shown in Figure 4. The excitation spectra mainly consist of two bands. One is a weak band with a maximum at 340 nm. The other is a strong broad band ranging from 400 nm to 480 nm, with a maximum at around 450 nm. These excitation bands arise from the absorption of the incident radiation by Ce^3+^ ions, which leads to the electronic transition from the ground state (2F_5/2_, 2F_7/2_) to the excited 5d levels. The emission spectra consist of a broad band ranging from 490 nm to 650 nm, with the maximum at around 520 nm, which are ascribed to the electronic transitions of Ce^3+^ from the lowest 5d level to the 4f ground state. After annealing at 1000 °C for 5 h in air atmosphere, 1.5 mol.% Ce^3+^ doped LuAG:Ce film exhibits the highest emission and excitation intensity. Improving the crystallinity and inhibiting the oxidation of Ce^3+^ could greatly enhance the photoluminescence properties. Upon annealing at 1500 °C for 5 h in CO atmosphere, 3.0 mol.% Ce^3+^ doped LuAG:Ce film exhibits the highest emission and excitation intensity. Compared with the 1.5 mol.% Ce^3+^ doped LuAG:Ce film annealed at 1000 °C in air atmosphere, the emission intensity of 3.0 mol.% Ce^3+^ doped LuAG:Ce film annealed at 1500 °C in CO atmosphere increases up to five times. Further increasing the Ce^3+^ doping concentration above 3.0 mol.% declines the emission intensity of LuAG:Ce films, which may be due to the effect of concentration quenching.

### 3.4. Chromaticity Coordinates and Color Purity of LuAG:Ce Films

**Figure 5:**
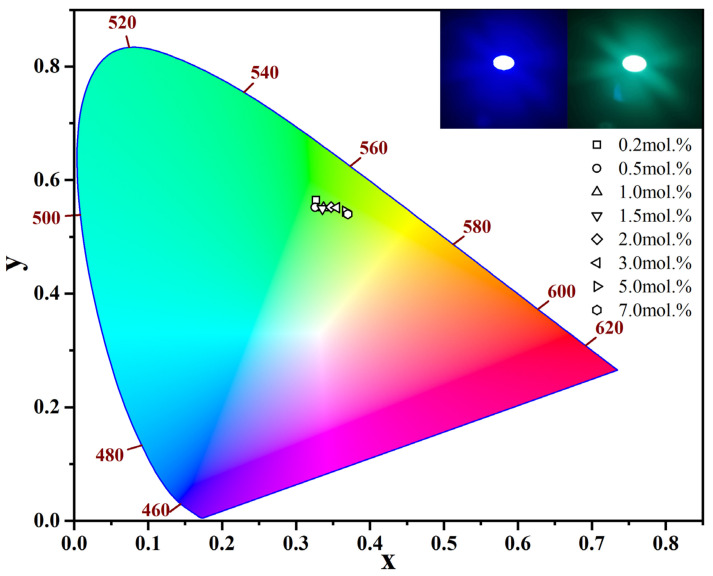
CIE color coordinates of 1500 °C CO atmosphere-annealed LuAG:Ce films with Ce^3+^ doping concentration ranging from 0.2 mol.% to 7.0 mol.% (excited at 448 nm). The insets are the digital images of blue lase (left) and lighting effects (right, lighting by the device composed of 3.0 mol.% Ce^3+^ doped LuAG:Ce film and a 0.5 W blue laser).

The corresponding chromaticity coordinates of LuAG:Ce films were obtained. The coordinates were calculated from the luminescence spectral data shown in Figure 4, and plotted in the 1931 chromaticity diagram, as shown in Figure 5. It is observed that the color changes from the yellowish green towards greenish yellow, as the Ce^3+^ doping concentration increases from 0.2 mol.% to 7.0 mol.%. After coupling the 3.0 mol.% Ce^3+^ doped LuAG:Ce film with a 0.5 W 450 nm blue laser, the formed device emits white light, as shown in Figure 5. The specific color coordinates and CCT of each LuAG:Ce film are presented in Table 1. As can be seen, the CCT shifts from the “cool white” region to the “neutral white” region, as the cerium concentration increases from 0.2 mol.% to 7.0 mol.% [25].

In order to further determine the photoluminescence properties of LuAG:Ce films, the color purity was investigated. The color purity can be calculated according to the following formula [26]:

(1) $$C=\frac{\sqrt{{\left(x-x_{i}\right)}^{2}+{\left(y-y_{i}\right)}^{2}}}{\sqrt{{\left(x_{d}-x_{i}\right)}^{2}+{\left(y_{d}-y_{i}\right)}^{2}}},$$

**Figure 6:**
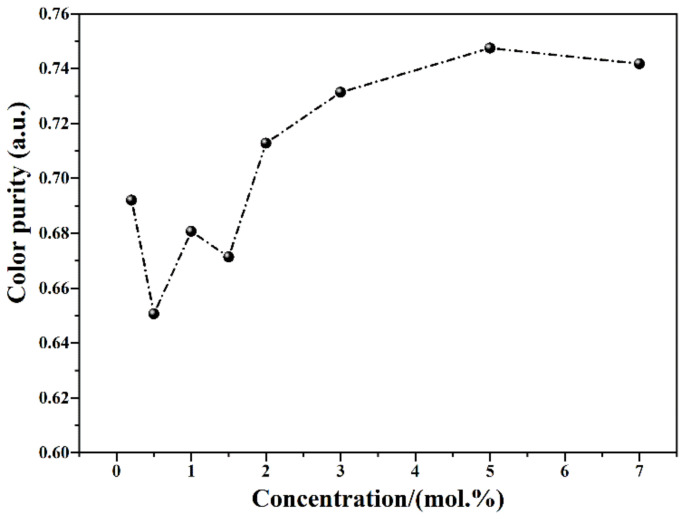
Color purity of 1500 °C CO atmosphere-annealed LuAG:Ce films with different Ce^3+^ doping concentrations.

Here, (x, y) are the CIE coordinates of LuAG:Ce film. (x_i_, y_i_) stands for the color coordinates of standard white light, while (x_d_, y_d_) are the color coordinates of the dominant wavelength. The calculated color purity of LuAG:Ce films is plotted in Figure 6. The highest color purity is up to 75%, which is ascribed to the 5.0 mol.% Ce^3+^ doped LuAG:Ce film.

### 3.5. The Appearance and Thickness of LuAG:Ce Films

**Figure 7:**
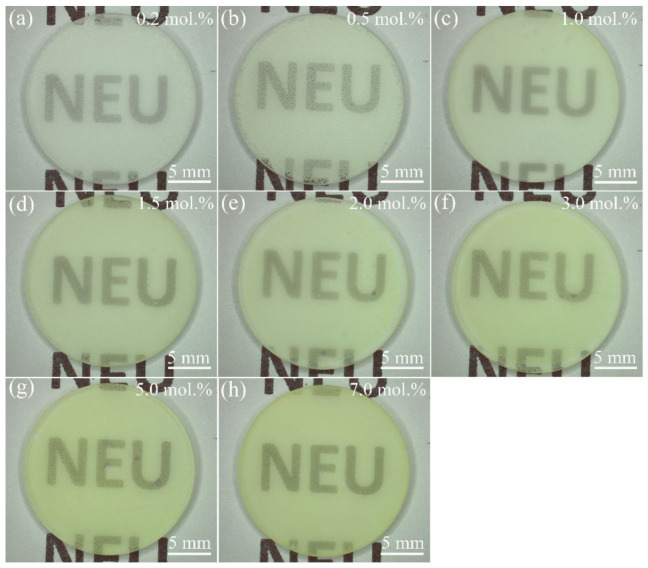
The appearance of 1500 °C CO atmosphere-annealed LuAG:Ce films with different Ce^3+^ doping concentrations: (**a**) 0.2 mol.%, (**b**) 0.5 mol.%, (**c**) 1.0 mol.%, (**d**) 1.5 mol.%, (**e**) 2.0 mol.%, (**f**) 3.0 mol.%, (**g**) 5.0 mol.%, (**h**) 7.0 mol.%.

**Figure 8:**
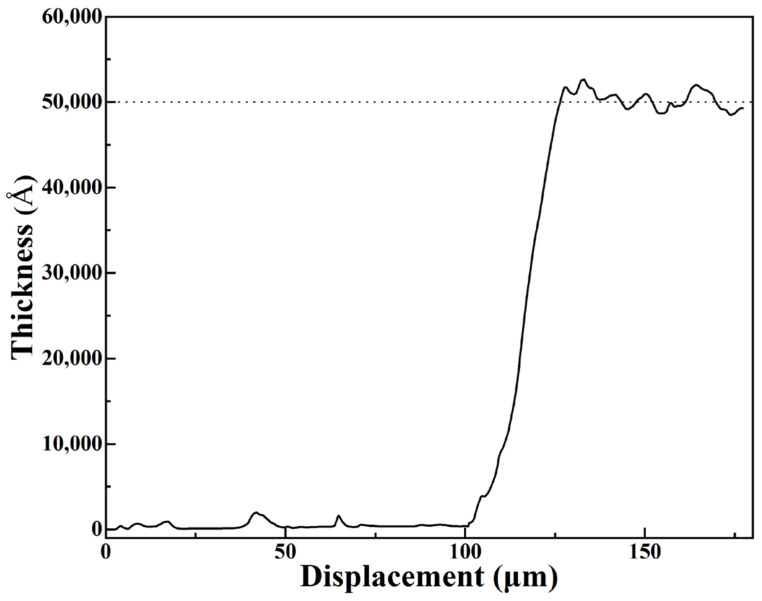
The thickness of 1500 °C CO atmosphere-annealed LuAG:Ce film with 3.0 mol.% Ce^3+^ doping concentration.

The appearance of 1500 °C CO atmosphere-annealed LuAG:Ce films with different Ce^3+^ doping concentrations are shown in Figure 7. The LuAG:Ce films are all translucent, thus the fonts “NEU” beneath the samples are clearly seen. As the Ce^3+^ doping concentration increases from 0.2 mol.% to 7.0 mol.%, the color of LuAG:Ce films changes from yellowish green to greenish yellow, which agrees well with the results of CIE color coordinates analysis. The thickness of LuAG:Ce film is also examined. Figure 8 shows the thickness of 3.0 mol.% Ce^3+^ doped LuAG:Ce film, which was annealed at 1500 °C for 5 h in CO atmosphere. As can be seen, 3.0 mol.% Ce^3+^ doped LuAG:Ce film has a thickness of around 5 μm.

## 4. Conclusions

LuAG:Ce films were successfully prepared by the ultrasonic spray pyrolysis process. The film thickness was around 5 μm. After annealing at 1000 °C in air atmosphere, LuAG:Ce films were well crystallized to the typical garnet structure. Improving the crystallinity and meanwhile inhibiting the oxidation of Ce^3+^ greatly enhanced the photoluminescence properties. After annealing at 1500 °C for 5 h in CO atmosphere, 3.0 mol.% Ce^3+^ doped LuAG:Ce film exhibited the highest emission intensity, which increased up to five times when compared with the emission intensity of the best LuAG:Ce film annealed at 1000 °C in air atmosphere. Coupling the 3.0 mol.% Ce^3+^ doped LuAG:Ce film with a 0.5 W 450 nm blue laser, the formed device successfully emitted white light.

## Figures and Tables

**Table 1:** CIE color coordinates and CCT of LuAG:Ce films.

Ce^3+^ Concentration	Coordinate (x)	Coordinate (y)	CCT (K)
0.2 mol.%	0.3268	0.5649	5693
0.5 mol.%	0.3258	0.5520	5714
1.0 mol.%	0.3370	0.5514	5499
1.5 mol.%	0.3355	0.5497	5527
2.0 mol.%	0.3473	0.5523	5307
3.0 mol.%	0.3547	0.5514	5171
5.0 mol.%	0.3670	0.5449	4941
7.0 mol.%	0.3700	0.5401	4878

## Data Availability

The data presented in this study are available from the corresponding authors upon reasonable request.
